# PB.24. How does volumetric breast density change with time?

**DOI:** 10.1186/bcr3729

**Published:** 2014-11-03

**Authors:** J Daniels, E Harkness, Y Lim, A Maxwell, J Morris, P Stavrinos, M Wilson, M Bydder, DG Evans, A Howell

**Affiliations:** 1 University of Manchester Medical School, Manchester, UK; 2 Centre for Imaging Sciences, Institute of Population Health, University of Manchester, UK; 3 Nightingale Centre and Genesis Prevention Centre, University Hospital of South Manchester, Manchester, UK; 4 University Hospital of South Manchester, Manchester, UK; 5 Manchester Breast Centre, Manchester Cancer Research Centre, University of Manchester, Christie Hospital, Manchester, UK

## Introduction

Breast density is a well-established independent risk factor for breast cancer, but risk may also relate to the rate at which breast density changes over time. Here we aim to establish the baseline rate of change in volumetric breast density in postmenopausal women undergoing mammographic screening.

## Methods

Data from 3,620 postmenopausal women attending two consecutive breast screening episodes were obtained from the PROCAS (Predicting Risk Of Cancer At Screening) study database. Women with current/previous breast cancer, current HRT users and those without digital mammographic raw data available were excluded. Volumetric breast density was obtained from digital mammograms taken at two consecutive screening episodes using Volpara software. Mean change in volumetric density was calculated, and its relationship with age, initial density and parity was assessed.

## Results

Mean volumetric breast density decreased from 6.43% at the initial screen to 5.76% at the subsequent screen, a mean decrease of 0.25% (percentage points) per year (*P *< 0.001). Fibroglandular volume showed no significant change, whereas breast volume increased by 30.13 cm^3^/year (*P *< 0.001), indicating that the decline in density between screens was predominantly due to an increase in nondense breast volume. Decline in breast density was greater with younger age and nulliparity.

## Conclusion

Postmenopausal women undergo a mean decrease in volumetric breast density of 0.25% (percentage points) per year, largely due to an increase in nondense breast volume. Establishing a baseline for volumetric breast density change over time will facilitate further work into whether variation in this pattern provides additional information in predicting breast cancer risk.

